# Biological Activity of Hydrophilic Extract of *Chlorella vulgaris* Grown on Post-Fermentation Leachate from a Biogas Plant Supplied with Stillage and Maize Silage

**DOI:** 10.3390/molecules25081790

**Published:** 2020-04-14

**Authors:** Dariusz Zielinski, Justyna Fraczyk, Marcin Debowski, Marcin Zielinski, Zbigniew J. Kaminski, Dorota Kregiel, Claus Jacob, Beata Kolesinska

**Affiliations:** 1 Institute of Organic Chemistry, Faculty of Chemistry, Lodz University of Technology, Zeromskiego 116, 90-924 Lodz, Poland; dbzdark@gmail.com (D.Z.); justyna.fraczyk@p.lodz.pl (J.F.); zbigniew.kaminski@p.lodz.pl (Z.J.K.); 2 Department of Environmental Engineering, Institute of Engineering and Environmental Protection, University of Warmia and Mazury in Olsztyn, Warszawska 117 a, 10-720 Olsztyn, Poland; marcin.debowski@uwm.edu.pl (M.D.); marcin.zielinski@uwm.edu.pl (M.Z.); 3 Department of Environmental Biotechnology, Faculty of Biochemistry and Food Sciences, Lodz University of Technology, Wolczanska 171/173, 90-924 Lodz, Poland; dorota.kregiel@p.lodz.pl; 4 Division of Bioorganic Chemistry, School of Pharmacy, Saarland University, D-66123 Saarbruecken, Germany; c.jacob@mx.uni-saarland.de

**Keywords:** microalgae, post-fermentation leachate, water extract, protein fraction, antioxidant activity, antibacterial and antifungal activity, nematicidal activity

## Abstract

Algae are employed commonly in cosmetics, food and pharmaceuticals, as well as in feed production and biorefinery processes. In this study, post-fermentation leachate from a biogas plant which exploits stillage and maize silage was utilized as a culture medium for *Chlorella vulgaris*. The content of polyphenols in hydrophilic extracts of the *Chlorella vulgaris* biomass was determined, and the extracts were evaluated for their antioxidant activity (DPPH assay), antibacterial activity (against *Escherichia coli*, *Lactobacillus* *plantarum*, *Staphylococcus aureus*, *Staphylococcus epidermidis*) and antifungal activity (against *Aspergillus niger*, *Candida albicans*, *Saccharomyces cerevisiae*). The use of the post-fermentation leachate was not found to affect the biological activity of the microalgae. The aqueous extract of *Chlorella vulgaris* biomass was also observed to exhibit activity against nematodes. The results of this study suggest that *Chlorella vulgaris* biomass cultured on post-fermentation leachate from a biogas plant can be successfully employed as a source of natural antioxidants, food supplements, feed, natural antibacterial and antifungal compounds, as well as in natural methods of plant protection.

## 1. Introduction

Marine organisms, including algae, are no less diverse than land vegetation. There are more than 30,000 different species of algae [1]. One of their unique features is the ability to live in almost any aquatic environment, including sweet, salty, saline, standing or flowing water. Microalgae compose a significant portion of algae species. About 9000 species of macroalgae have been identified in oceans around the world [2], although this is thought to be only a small fraction of the total number. Algae are divided into three main groups [3]: green algae (Chlorophyta); red algae (Rhodophyta) and brown algae (Phaeophyta) [4]. Algae have a wide range of applications and huge potential for many other uses, including in medicine [5,6,7] and as a heavy metal bioindicator for testing ecosystem pollution. Research conducted around the world indicates that algae, including macroalgae, are characterized by high antibacterial [8,9,10,11,12], antifungal [13] and/or anticancer activities [14,15,16,17]. Their varied biological activity is due to the presence of an arsenal of bioactive primary and secondary metabolites, such as polyphenols, carotenoids, saponins, flavonoids, tannins and cardiac glycosides [18].

*Chlorella* is a green algae that lives in both salt and fresh water. Around 2000 tons are produced in 70 countries across the world [19]. It is estimated that 60% of the *Chlorella* organism comprises of proteins. Other constituents include fats, fibers, carbohydrates, and a full range of vitamins and minerals, thanks to which supplements based on *Chlorella* sp. can be helpful in the treatment of many serious diseases. The main minerals and vitamins found in *Chlorella* sp. include iron, calcium, potassium, zinc, manganese, selenium, magnesium, folic acid lutein, group B vitamins, group E vitamins and beta carotene, which have a beneficial effect on the skin and eyesight [20,21,22,23]. Many different compositions containing *Chlorell*a sp. are employed in the treatment of various ailments and diseases, and to detoxify the body by removing heavy metals. *Chlorella vulgaris* can be used as a food supplement and/or additive [24,25,26], a colorant (after carotenogenesis) and a food emulsifier [27]. Despite its diverse biological activity and health benefits, *Chlorella vulgaris* is classed as a nutraceutical, due to the lack of official legislation regarding the quality and requirements for microalgae [28,29,30].

Waste biomass of microalgae after the extraction of valuable chemical compounds can also be utilized for the production of biofuels. Depending on the composition of the biomass, it can be used for the production of liquid biofuels (bioethanol, biobutanol, biodiesel and biooil) [31,32] or gas biofuels (biomethane, biohydrogen, synthesis gas, etc.) [33,34,35]. However, an efficient technology for producing biofuels from waste algae biomass has not yet been fully developed, and it is therefore necessary to combine various biological and thermochemical processes in order to obtain the highest possible efficiency [36,37,38,39,40,41]. *Chlorella vulgaris* has often been used in biorefining processes [42,43,44,45,46,47]. In classic biorefinery processes, after the isolation of valuable chemical compounds, algae biomass residues may be used to produce biofuels and energy.

In this study, we investigated the possibility of cultivating *Chlorella vulgaris* microalgae using a culture medium based on digestate effluent. The effluent was sourced from a biogas plant operated on the technical scale, fed with distillery and maize silage. We also tested the effects of the culture medium on the antioxidant and antimicrobial activities of the algae. The biological activity of the algae was assessed in terms of its suitability for use in feed and as a natural antifungal, antibacterial and nematicidal agent against *Steinernema feltiae* in natural plant protection products.

## 2. Results and Discussion

The studies began with quantitative determination of the polyphenols (which are secondary metabolites found in algae) in the hydrophilic fraction of the algae *Chlorella vulgaris*, based on a method using Folin–Ciocalteu reagent. So far, two biosynthesis mechanisms have been identified, known as the shikimic and acetate-malonic acid pathways [48]. Polyphenols are antioxidants, which together with ascorbic acid, carotenoids and tocopherols protect the human body against oxidative stress. In the aqueous extract of algae biomass (the hydrophilic fraction), the content of polyphenols expressed as pyrogallol was found in reasonable amounts i.e., 0.77 mM (the average of three replicates) (Table 1). The polyphenols were also found in the protein fraction obtained after protein precipitation by trichloroacetic acid (TCA) in significant amount i.e., 0.33 mM. However, in the combined protein pellets obtained after another five centrifugations of the initial supernatant (after the first centrifugation of the protein fraction), the content of polyphenols calculated as pyrogallol was reduced to 0.16 mM. The positive result for polyphenols in the protein fraction may be due to the presence of tyrosine in proteins, which gives a positive test for polyphenols [49], or the occurrence of protein–polyphenol complexes.

The content of polyphenols indicates that the hydrophilic fraction of *Chlorella vulgaris* algae is characterized by satisfactory antioxidant activity. This means that even when leachate from biorefinery processes is exploited as a medium, the obtained algae show high potential as raw materials for utilization in the pharmaceutical industry, cosmetics or food supplements.

The protein content of the aqueous extracts was determined according to the Barbino and Laurenco procedure [50]. In the water fractions extracted from the algae biomass, the protein content ranged from 10%–14%; the average quantity of five *Chlorella vulgaris* breeding batches accounted 12.8%. According to the literature, the total protein content in algae can reach 20%–45% [51,52] and varies according to growth conditions. In microorganisms, proteins play multiple roles. Approximately 20% of the total proteins are bound to the cell wall, more than 50% constitute internal proteins and 30% migrate in and out of the cell [53]. Thus, the protein content determined in *Chlorella vulgaris* did not differ substantially from the typical content found in this type of organism, despite the fact that the algae were grown with leachate from biorefinery processes.

**Figure 1:**
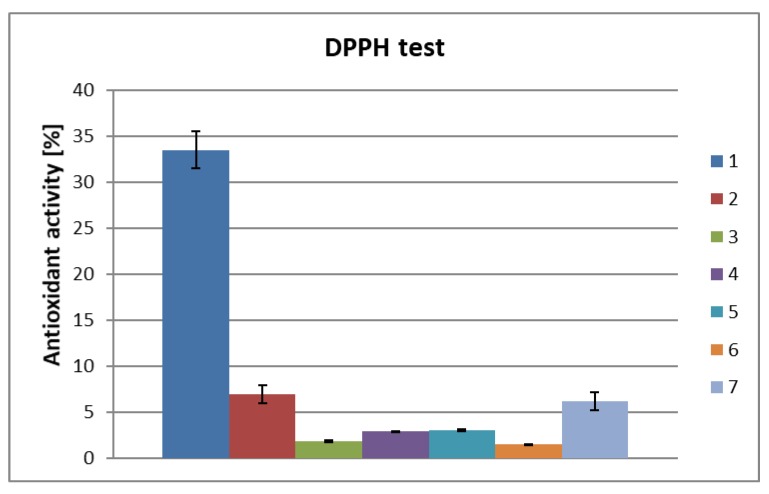
Antioxidant activity of hydrophilic (water and protein) fractions obtained from algae biomass: (1) ascorbic acid 1 nM (0.17 mg/mL); (2) water extract first repetition, c = 0.5 mg/mL; (3) water extract, second repetition, c = 0.2 mg/mL; (4) water extract, third repetition, c = 0.2 mg/mL; (5) protein fraction obtained from sample 2 treated with TCA and then centrifuged once, c = 0.5 mg/mL; (6) protein fraction obtained from sample 4 treated with TCA and then centrifuged once, c = 0.2 mg/mL; (7) combined protein fraction after another four centrifugations of the supernatant obtained from sample 6, c = 0.2 mg/mL.

In order to assess the antioxidant properties of the hydrophilic fractions obtained from biomass of *Chlorella vulgaris*, the DPPH assay was performed employing ascorbic acid as a positive control (Figure 1).

The algae biomass water extract at a concentration of 0.5 mg/mL exhibited an antioxidant activity of 7% (Figure 1), which is five times lower than that of the ascorbic acid which was employed as a positive control. Reducing the concentration of the aqueous extract to 0.2 mg/mL further lowered the antioxidant activity to 2%–3%. A decreased antioxidant activity to about 3% was observed for proteins obtained as a result of single centrifugation of the TCA-treated solution (concentration of protein components = 0.5 mg/mL), while the sample with a concentration of 0.2 mg/mL exhibited 1.5% of the radical scavenging activity. After first precipitation, the supernatant was centrifuged four more times to release additional amount of the protein fractions. The combined protein fractions (after subsequent centrifugations) demonstrated an antioxidant activity 6% of ascorbic acid standard at a sample concentration of 0.2 mg/mL (Figure 1; bar 7). It appears that this was caused by the precipitation of protein-polyphenol complexes during subsequent centrifugation, since the protein fractions provided a positive result for the Folin–Ciocalteu test (Table 1).

**Figure 2:**
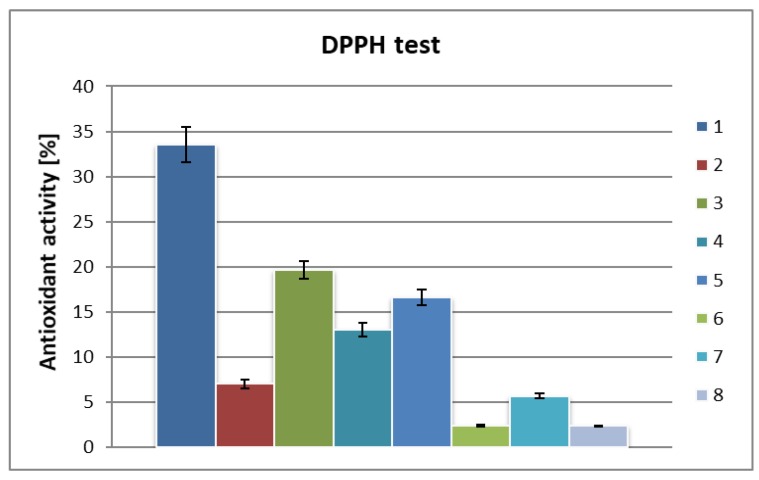
Total antioxidant activity of hydrophilic fractions from algae biomass separated by ultrafiltration: (**1**) ascorbic acid 1 nM (0.17 mg/mL); (**2**) water extract (before separation) c = 0.5 mg/mL; (**3**) residue on the 50 kDa filter, c = 0.5 mg/mL; (**4**) residue on the 10 kDa filter, c = 0.5 mg/mL; (**5**) residue on the 5 kDa filter, c = 0.5 mg/mL; (**6**) filtrate after separation on the 50 kDa filter, c = 0.5 mg/mL; (**7**) filtrate after separation on the 10 kDa filter, c = 0.5 mg/mL; (**8**) filtrate after separation on the 5 kDa filter, c = 0.5 mg/mL.

The water extract from the *Chlorella vulgaris* algae was subjected to a separation test, exploiting an ultrafiltration technique utilizing filters of 50,000, 10,000 and 5000 Da and for each separation fresh extract was used. The supernatants (filtrates) obtained as a result of filtration, as well as the residue deposited on the filter, were assessed in terms of their antioxidant activity (Figure 2).

The residues isolated by filtration were found to have significantly higher radical scavenging activity (i.e., two to three fold) compared to the initial aqueous extract (Figure 2; compare bars 3,4,5 vs. bar 2). The increase in activity was 2–3 times higher. The influence of the filter pore size on the cut-off level was also surprising. The residues on the 50 kDa and 5 kDa filters were more active than those on the 10 kDa filter. In all cases, antioxidant activity was evaluated employing the same concentrations, so the observed variation in activity is not the result of different concentrations. Compounds isolated from the 50 kDa filter (c = 0.5 mg/mL) demonstrated high antioxidant activity, at almost 20%, which is 58.5% of the value observed for ascorbic acid. On the other hand, the compounds deposited on the 5 kDa filter (c = 0.5 mg/mL) had an activity of 16.6%, which is 49.5% of the activity of the ascorbic acid. The compounds deposited on 10 kDa filter presented the lowest activity of 13% (corresponding to 38.8% of the ascorbic acid activity). The result obtained can suggest the presence in residues isolated on 50 kDa and 5 kDa filters of at least to different components active as antioxidants with strongly diversified molecular size. Significantly lower antioxidant activity was found for filtrates after separation on all filters used. The values for antioxidant activity ranged from 2.3%–5.6% (compared to 33.5% for ascorbic acid) (Figure 2; bars 6, 7, 8). In all cases, a concentration of 0.5 mg/mL was used for both the compounds embedded in the filters and those which had passed through the filters. In the case of the filtrates, the highest antioxidant activity (5.6%) was observed for the solution of compounds which had passed through the 10 kDa filter. It is worth noting that the compounds deposited on this filter also showed the lowest antioxidant activity.

**Figure 3:**
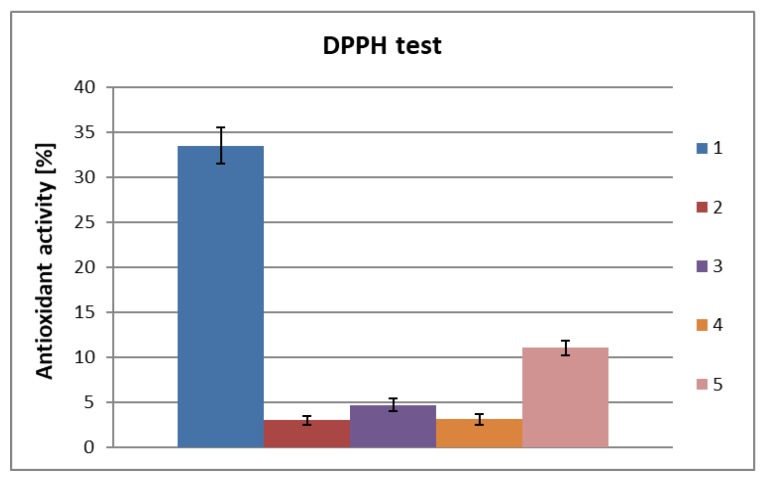
Total antioxidant activity of protein fractions obtained after treatment of aqueous biomass extract of *Chlorella vulgaris* with TCA separated by ultrafiltration: (**1**) ascorbic acid 1 nM (0.17 mg/mL); (**2**) protein fraction before separation (obtained by precipitation by TCA and a single centrifugation), c = 0.5 mg/mL; (**3**) protein filtrate after ultrafiltration on a 50 kDa filter, c = 2 mg/mL; (**4**) protein filtrate after ultrafiltration on a 10 kDa filter, c = 2 mg/mL; (**5**) protein filtrate after ultrafiltration on a 5 kDa filter, c = 1 mg/mL.

The protein fraction precipitated from the aqueous fraction using TCA was also ultrafiltrated, leading to protein/peptide solutions with different molar weights (Figure 3). The same range of filters was used.

It was found that the protein filtrate ultrafiltered on a 50 kDa filter at a concentration of 2 mg/mL exhibited slightly higher activity than the inseparable protein fraction at the same concentration (Figure 3; bars 2 and 3). An antioxidant activity of 4.7% was observed for the protein filtrate. Filtration with a 10 kDa filter allowed a protein fraction to be obtained with identical antioxidant activity as that of the unseparated protein fraction (Figure 3; bars 2 and 4). The highest antioxidant activity (11.1%) was observed for the protein/peptide fraction after filtration using the 5 kDa filter at a concentration of 1 mg/mL. In this case, a four-fold increase in antioxidant activity was observed compared to the protein fraction before separation (Figure 3; bars 2 and 5). These results indicate that low molecular weight protein/polypeptide compounds are associated with antioxidant activity.

**Figure 4:**
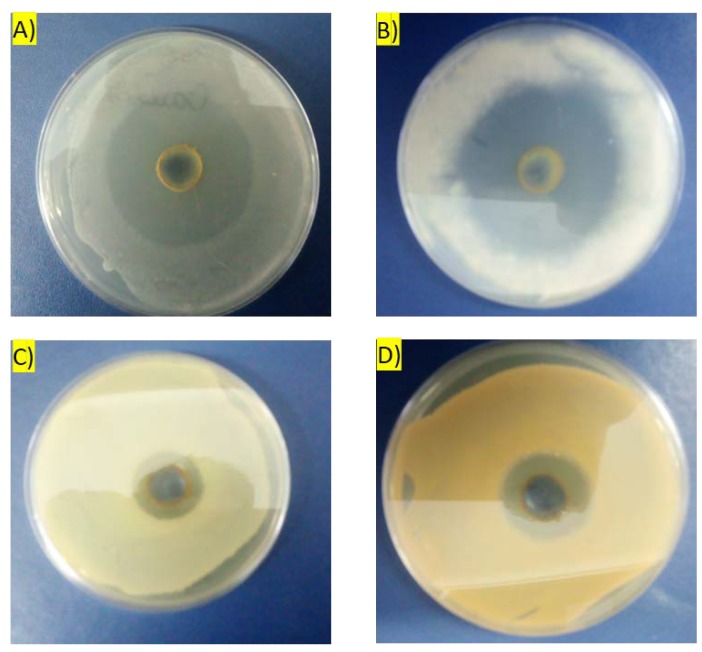
Antimicrobial activity of aqueous extract of *Chlorella vulagris*: (**A**) *Aspergillus niger*, (**B**) *Candida albicans*, (**C**) *Escherichia coli*, (**D**) *Staphylococcus aureus*.

In the next stage of the research, we investigated the antimicrobial properties of aqueous extract from *Chlorella vulgaris* biomass. A 150 mg/mL solution was used. Antimicrobial activity was tested against *A. niger*, *C. albicans*, *E. coli* and *S. aureus* (Figure 4).

It was found that an aqueous extract with a concentration of 150 mg/mL exhibited antimicrobial activity against all tested organisms. The diameters of the inhibition zones were as follows: *A. niger*—51 mm; *C. albicans*—47 mm; *E. coli*—24 mm; *S. aureus*—25 mm. Therefore, the aqueous biomass extract of *Chlorella vulgaris* demonstrated higher antifungal activity in comparison to antibacterial action. It was documented in the literature that cell extracts of various algae, namely *Ulva* sp. and *Chlorella* sp. exhibit antifungal activity in vitro [54]. In our study, the aqueous extract of *C. vulgaris* also showed antibacterial activity against both Gram-negative (*E. coli*) and Gram-positive (*S. aureus*) bacteria. In other research, extracts of green unicellular algae showed the pronounced antagonistic activity against numerous opportunistic and pathogenic bacteria [55,56]. In this connection, our results confirm and reveal the potential of *C. vulgaris* extracts for the production of natural fungicides and bactericides. In addition, the zones of inhibition found under the treatment with aqueous extracts of Chlorella vulgaris indicate their high antibacterial activity. Literature data show [57,58] that the use of organic solvents (methanol, acetone, ethanol and chloroform) to extract biologically active compounds from microalgae grown in media useful in biorefinery processes leads to much smaller inhibition zones.

The encouraging results for antimicrobial activity prompted us to investigate the minimum inhibitory concentration (MIC) of both the aqueous extract of *Chlorella* sp. biomass and of the protein fraction obtained as a result of protein precipitation by TCA of the hydrophilic fraction (Table 2). The microorganisms used were *L. plantarum*, *S. epidermidis*, *E. coli*, *C. albicans* and *S. cerevisiae*.

The *Chlorella vulgaris* biomass water extract demonstrated no activity against *S. cerevisiae*, but significant activity was observed against *C. albicans* with an MIC in the range of 37.5–75 mg/mL. The components of the aqueous extract presented relatively low activity against *E. coli* and *S. epidermidis* with the MIC value of 150 mg/mL. Significantly higher antimicrobial activity was observed against *L. plantarum* with an MIC in the range of 37.5–75 mg/mL. The highest antimicrobial activity was observed for the protein fraction obtained by TCA from the aqueous extract with an MIC value of 32.5–65 mg/mL against *E. coli* and 32.5 mg/mL against the fungi, *C. albicans* and *S. cerevisiae*, respectively. This indicates that protein/polypeptide compounds were responsible for the antifungal activity, because the full aqueous extract demonstrated lower or no antifungal activity. The protein fraction isolated from the *C. vulgaris* biomass exhibited excellent antimicrobial activity against *L. plantarum* and *S. epidermidis* with the MIC values of 16.25 mg/mL.

**Figure 5:**
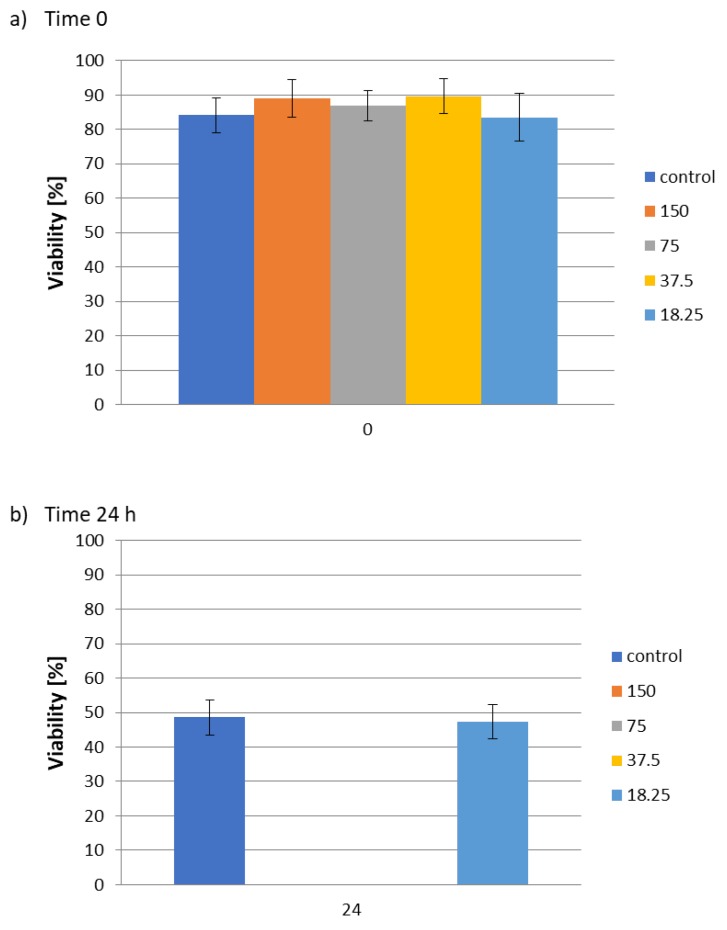
Influence of hydrophilic components (water extract) of *C. vulgaris* biomass on *S. feltiae* viability: (**a**) time 0; (**b**) after 24 h of incubation.

Attempts were also made to assess the activity of the aqueous extract of *C. vulgaris* biomass against *Steinernema feltiae* belonging to the family of *Entomopathogenic nematodes* (EPN). EPN live in an infected host and are therefore referred to as parasitic. Moreover, EPN are active on many different types of soil insects, such as moth larvae, butterflies, flies, beetles, grasshoppers and crickets, and can directly threaten higher organisms. On the other hand, EPN can also be employed for the biological control of harmful insects [59]. Preliminary results in the literature indicate that *C. vulgaris* is active against the nematode *Meloidogyne arenaria* and some ectoparasites [60]. *C. vulgaris* activity against root parasites could therefore be used in ecological methods of plant protection. The aqueous extract of *Chlorella* sp. biomass was evaluated for nematicidal activity against *Steinernema feltiae* with concentrations ranging from 18.25–150 mg/mL (Figure 5).

The viability of *Steinernema feltiae* was assessed after 24 h of incubation. At concentrations of 37.5, 75 and 150 mg/mL, the aqueous extract of *C. vulgaris* biomass caused complete mortality of the nematode *Steinernema feltiae*. At a concentration of 18.25 mg/mL, no effect on nematode survival was observed, which was close to the control value.

## 3. Materials and Methods

The species of *C. vulgaris* (UTEX 2714) used in the study was obtained from the UTEX algae culture collection at the University of Texas in Austin (USA). An initial concentration of 32.5 mg/mL of microalgae biomass was obtained in the photobioreactor. The culture medium was obtained on the basis of post-fermentation leachate from a biogas plant operated on a technical scale, fed with stillage and maize silage. The technological parameters of the biogas plant were as follows: concentration of anaerobic sludge in the digesters—5.0 g/L; operation at 40 °C; load of organic compounds—2.4 g/L, hydraulic stop time—40 days.

The post-fermentation effluents were subjected to processing prior to their introduction into the photobioreactors, then distilled at 100 °C using distillation flasks with an active volume of 200 mL. The distillation flasks were connected to a spiral cooler, where the digestate effluent vapors were condensed. The cooling medium was cold tap water. The resulting distillate was collected and stored in a 200 mL flask. The aim was to remove solid suspensions and color, obtaining the component of the culture medium that contained nutrients in the dissolved phase, as well as hygienization of the tested post-fermentation leachate. Basic parameters characterizing the post-fermentation leachate are presented in Table 3.

Due to the high concentration of organic compounds and the color of the digestate, the volume introduced into the culture medium was 50% of the total active volume of the photobioreactors. The remainder was supplemented with deionized water.

Preliminary results indicate that *Chlorella* biomass grown using synthetic media such as B−11 did not differ significantly in biomass growth rate or photosynthetic activity from biomass grown on properly prepared effluent from a biogas plant. During *Chlorella vulgaris* cultivation using post-fermentation leachate obtained average 2400 ± 120 mg/L dry organic mass. When typical medium B−11 was used dry organic mass obtained 2450 ± 140 mg/L dry organic mass. There were not statistical differences between cultures cultivated on B−11 medium and post-fermentation leachate. The effluent was obtained from stabilized post-fermentation sludge. Minerals such as nitrogen were key to growing algae. Sludge with an organic fraction below 60% is considered to be biologically stable.

It has also been found that biomass can even grow better, and the culture is more homogeneous because other algae species do not tolerate leachate as well [61,62,63].

### 3.1. Research Stand

**Figure 6:**
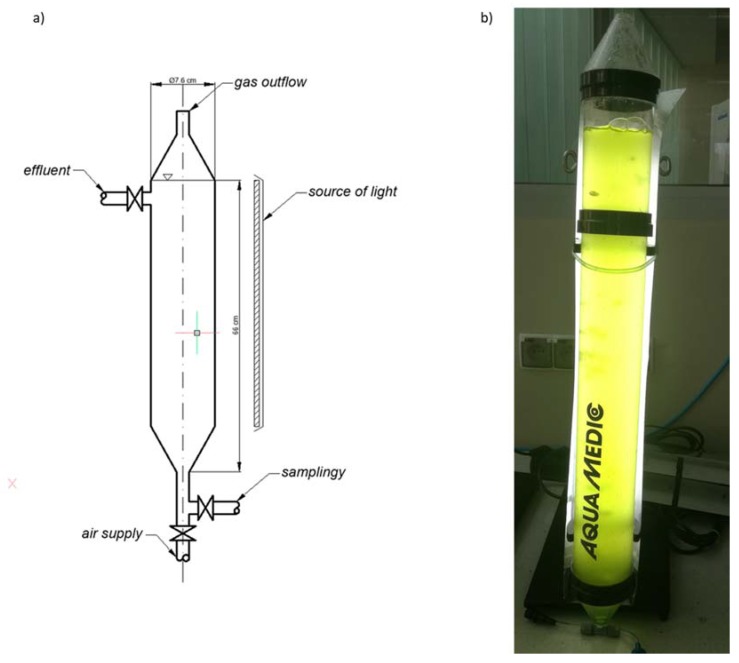
Scheme of photobioreactor used for breeding the algae (**a**). Photo of the photoreactor used in the research (**b**).

Vertical tubular reactors with an active volume of 2.5 L were used to culture the microalgae biomass (Figure 6). The culture was grown under continuous lighting (fluorescent lamp, colored temperature 9000 K), light intensity 12,000 lx, culture temperature 23 °C ± 0.5 °C. The contents of the columns were constantly aerated by compressed air supplied from below the reactors using Mistral 200 peristaltic pumps (Whale Tankers, Ravenshaw, United Kingdom) with a capacity of 200 dm^3^/h. This ensured the introduction into the system of carbon dioxide and effective mixing of the algae culture.

Technical parameters of a single test set:

Overall height        H_total_ = 72 cm

Active height        H_active_ = 66 cm

Inner diameter        D_inner_ = 7 cm

Active chamber volume   V_o_ = 2.5 L

### 3.2. Analytical Methods for Assessing the Microalgae Culture

The cultivation time was 14 days, after which we determined the concentration of microalgae biomass and the concentration of monitored pollution indicators in the culture medium. The samples were centrifuged for 10 min using a laboratory centrifuge (MPW−251, Donserv, Warsaw, Poland) with a rotational speed of 15,000× *g*. The retentate content was then determined by the dry mass method. The supernatant concentration of N-NH_4_ was measured by Hach–Lange cuvette tests (Duesseldorf, Germany) using a DR 5000 spectrophotometer (Hach–Lange) with a HT 200 s mineralizer (Hach–Lange). At the start and end of the experiment, both the leachate and the culture medium were tested for biochemical oxygen demand using the Oxi-top control system (WTW, Weilheim, Germany) and chemical oxygen demand P-PO_4_, P_total_, N_total_ using a DR 5000 spectrophotometer (Hach–Lange) with an HT 200s mineralizer (Hach-Lange). The pH was determined using a VWR 1000 L pH meter (Gdansk, Poland). The intensity of light supplied to the operated PBRs was measured with a Lux-meter NL−100 (Hanna, Sonopan, Bialystok, Poland). Qualitative phytoplankton analysis was performed by microscopic analysis at a magnification of 1.25 × 10 × 40 or 1.25 × 10 × 100, as well as using an algae biomass BB Moldanke analyzer (Schwentinental, Germany).

### 3.3. Disintegration of Cell Walls

A suspension of *Chlorella vulgaris* in water was frozen in liquid nitrogen then dehydrated by freeze-drying (FreeZone 1 Labconco Freeze Dryers) (A.G.A. Analytica, Warsaw, Poland). The lyophilizate was stored at a temperature of −20 °C. In each disintegration cycle, 1.5 g of dry lyophilizate from the algae biomass was used. The dry mass was suspended in 20 mL distilled water. The suspension was then sonicated (100 amplitude, pulse, time 5 min) using a homogenizer. After homogenization, the samples were again freeze-dried. The lyophilizate was stored at a temperature of −20 °C.

### 3.4. Isolation of the Hydrophilic Fraction

Homogenized lyophilizate from algae biomass (1 g) was suspended in 30 mL of distilled water. The suspension was stirred for 24 h in a water-ice bath (temp. 0–5 °C). The suspension was then centrifuged (Universal 3200R Hettrich Zentrifugen, 15,000× *g*, 40 min, 4 °C) (Labo Baza, Jelonek, Poland). The pellet was suspended in 4 mL of 1 mM NaOH solution, shaken for 30 min and centrifuged again (15,000× *g*, 40 min, 4 °C). The aqueous fractions were combined and lyophilized. Both the lyophilizate and insoluble algae biomass residues were stored at −20 °C. The experiment was repeated at least three times.

### 3.5. Isolation of Protein/Polypeptide Fraction

We used a modified version of the method described by Barbino and Laurenco [50]. The lyophilizate of the hydrophilic fraction (0.5 g) was suspended in distilled water (10 mL). Then, 25 mL of a 25% aqueous trichloroacetic acid (TCA) solution was added to the solution. The solution was shaken vigorously for 5 min. The mixture was then incubated for 1.5 h at 0–5 °C. After incubation, the mixture was centrifuged (15,000× *g*, 40 min, 4 °C). The precipitate was dissolved in 10 mL of 0.1 mM NaOH aqueous solution and lyophilized. The supernatant was centrifuged a further four times under the same conditions. The resulting precipitates were combined and dissolved in 10 mL of 0.1 mM NaOH aqueous solution and lyophilized. The supernatant residue was discarded. The lyophilizate was stored at −20 °C. The experiment was repeated at least three times.

### 3.6. Ultrafiltration

The protein pellet and hydrophilic fraction were separated using the ultrafiltration method. For each experiment, a fresh sample was suspended in distilled water and centrifuged (15,000× *g*, 8 h, 4° C) using an ultrafiltration tube (Vivaspin 50,000, 10,000, 5000 MWCO) (Merck KGaA, Darmstadt, Germany). The residue on the filter and the filtrate were collected, deep frozen in liquid nitrogen then lyophilized. The dried samples were stored at −20 °C. The experiment was repeated at least three times.

### 3.7. Determination of Protein Content by Lowry’s Method

**Figure 7:**
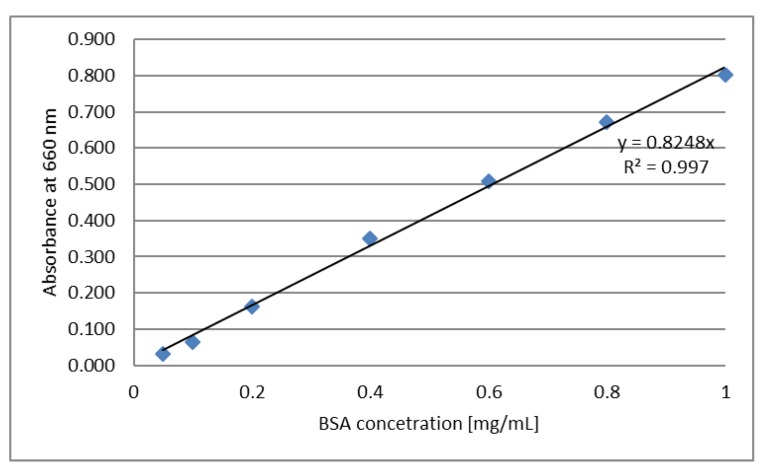
Calibration curve—absorbance dependence on Bovine Serum Albumin (BSA) concentration.

The total protein content was determined using the standard Lowry′s procedure [64]. Preparation of analytical reagents: (A solution) 50 mL of 2% sodium carbonate was mixed with 50 mL of 0.1 mM NaOH solution; (B solution) 10 mL of 1.56% copper sulphate solution was mixed with 10 mL of 2.37% sodium potassium tartrate solution; (C solution) 2 mL of B solution was mixed with 100 mL A solution. Preparation of Lowry′s assay: 0.2 mL of a sample dissolved in water was mixed with 2 mL C solution. The solution was incubated at room temperature for 10 min. Then, 0.2 mL of 1 M Folin–Ciocalteau solution was added and the sample was incubated at room temperature for an additional 30 min. After incubation, absorbance was measured at a wavelength of 660 nm (Orion AquaMate 8000 UV-VIS Spectrophotometer, Thermo Scientific, Anchem, Warsaw, Poland). Distilled water was used as a control. The calibration curve was determined using Bovine Serum Albumin (BSA) in a concentration range from 0.05 to 1 mg/mL (Figure 7). The experiment was repeated at least three times.

### 3.8. Determination of Polyphenol Content

**Figure 8:**
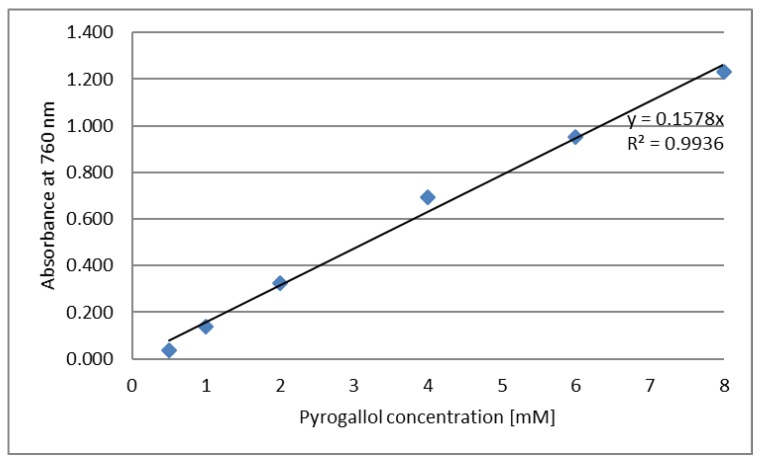
Calibration curve—absorbance dependence on pyrogallol concentration.

Total polyphenol content was determined using Folin–Ciocalteu reagent [65]. To prepare the samples for the polyphenols assay, 40 µL of the tested sample was dissolved in water, then 800 µL of Folin–Ciocalteau solution was added and mixed carefully. The obtained solution was incubated at room temperature for 5 min. Then, 800 µL of 7% w/v sodium carbonate aqueous solution and 360 µL water were added and the mixture was vortexed. The solution was incubated at room temperature for 2 h. After incubation, absorbance was measured at a wavelength of 760 nm (Orion AquaMate 8000 UV-VIS Spectrophotometer, Thermo Scientific). Distilled water was used as a control. The calibration curve was determined using pyrogallol at a concentration in the range of 0.5–8 mM (Figure 8). The experiment was repeated at least three times.

### 3.9. Antioxidant Activity

The 2,2-diphenyl-1-picrylhydrazyl (DPPH) assay was used to determine the antioxidant activity of the samples [66], with a 0.5 mM solution of DPPH in ethanol. To 20 µL of a sample dissolved in water was added 1500 µL of DPPH solution. The sample was incubated at room temperature for 30 min. After incubation, absorbance was measured at a wavelength of 517 nm. As an antioxidant standard, 1 nM solution of ascorbic acid was used.

Total antioxidant capacity was calculated using the formula:

$$I=\left[\frac{A0-\left(At-As\right)}{A0}\right]\;\times \;100\%$$

where:

A_0_–absorbance of DPPH solution
A_t_–absorbance of the tested sample after 30 min incubation with DPPH solution
A_s_–absorbance of the tested sample without DPPH solution.

### 3.10. Microbiological Tests

#### 3.10.1. Inhibition Zone Plate Assay

The inhibition zone plate assay was performed on Petri dishes with agar media, appropriate for selected microorganisms: MEA (Sigma-Merck Merck KGaA, Darmstadt, Germany) for fungi, and TSA (Sigma-Merck) for bacteria. The following microorganisms were tested: *Aspergillus niger* LOCK0440, *Candida albicans* ATCC10231, *Staphylococcus aureus* ATCC6538 and *Escherichia coli* ATCC8739. The microorganisms were subcultured previously on agar media. One or two isolated colonies of the tested microorganisms were touched using a sterile cotton swab, suspended in 5 mL of sterile saline medium and vortexed well until a uniform suspension was obtained. The turbidity of the suspension was measured using a DEN−1 densitometer (Merck, Darmstadt, Germany). The turbidity of the suspension was adjusted to a 0.5 McFarland standard by adding more microorganism if the suspension was too light or diluting with sterile saline if the suspension was too heavy. The suspension was prepared before inoculating the microorganisms on the agar plate. To inoculate the agar plates, a sterile cotton swab was dipped into the suspension and streaked over the surface of the agar plates. This procedure was repeated three times; each time, the plate was rotated approximately 60 °C to ensure even distribution of the inoculum. The plates were then allowed to dry at room temperature for 5 min before cutting wells. Each well was 14 mm in diameter, and the cut-out of the agar was removed using a sterile needle. After this, each well was filled with 400 μL algae biomass extract at a concentration of 150 mg/mL. The antimicrobial activity of algae extract was visible as forming a zone of growth inhibition after incubation at temp. 37 °C (48 h for bacteria, 5 days for fungi) with tested microbial strains. The experiments were repeated at least three times.

#### 3.10.2. Minimum Inhibitory Concentration Assay

The following microorganisms were used: *Lactobacillus* (MRS), *Staphylococcus epidermidis* (Luria Broth), *Escherichia coli* (Luria Broth), *Candida albicans* (Sabouraud Dextrose Broth), *Saccharomyces cerevisiae* (YPD Broth). Micro-organisms previously amplified with the appropriate media were transferred by a loop into a flacon containing 10 mL of 0.9% NaCl solution, obtaining a solution with absorbance in the range of 0.8–1 (solution D) (measurement at 600 nm). Then, 0.15 mL of solution D was added to 14.85 mL of the appropriate medium. The resulting solution was mixed well and transferred to a 96 well plate, 75 mL per well. The wells were completed with 75 µL of test solutions in a dilution series (aqueous extracts of algae biomass: 300; 150; 75.5; 37.5; 18.75; 9.38; 4.69; 2.35 mg/mL; the protein fraction after TCA precipitation and the first centrifugation: 130; 65; 32.5; 16.25; 8.13; 4.07; 2.04; 1.02 mg/mL). The media for each microorganism were used as controls. After 24 h of the incubation at 37 °C and 5% CO_2_ (incubation chamber: Thermo Scietific, Model 310), the wells were inspected for the propagation of microorganisms.

The following bacteria and yeast used in the experiments were sourced from Leibniz Institute DSMZ German Collection of Microorganisms and Cell Cultures GmbH and stored at −80 °C (Institute of Bioorganic Chemistry, University of Saarland, Saarbruecken, Germany): *Lactobacillus*, *Staphylococcus epidermidis*, *Escherichia coli*, *Candida albicans*. The experiments were repeated at least three times.

### 3.11. Nematode Activity Assay

The model of the nematode *Steinernema feltiae* was purchased in the form of powder from Sautter und Stepper GmbH (Ammerbuch, Germany) and stored in the dark at 4 °C. A homogeneous mixture was prepared by dissolving 200 mg of nematode powder in 50 mL of phosphate buffered saline (PBS pH 7.4). The solution was shaken and incubated for 30 min at room temperature under visible light. The viability of *Steinernema feltiae* was observed under a microscope (TR 200, VWR International Belgium). Above 80% viability of nematodes in the sample was considered as a prerequisite for each experiment.

To each of the 96 wells, 80 µL of PBS pH 7.4 was added, followed by 10 µL of nematode suspension and 10 µL of the samples. The samples of water extract algae biomass were used at concentrations of 150, 75, 37.5 and 18.37 mg/mL. PBS and ethanol were used as negative and positive controls. Living and dead nematodes were counted under a microscope prior to treatment. The 96-well plate with samples was incubated for 24 h at room temperature in the dark. After this time, 50 µL of lukewarm water (50 °C) was added to each well, to stimulate the nematodes prior to counting. Viability was calculated using the formula:

V = (V_l_/V_N_) × 100%

V–viability [%]
V_l_–number of living nematodes
V_n_–number of all nematodes

The experiments were repeated at least three times.

### 3.12. Statistical Methods

Each variant of the experiments was carried out in triplicate. Statistical analysis of the results was based on STATISTICA 10.0 PL (StatSoft Polska, Cracow, Poland). Our hypothesis regarding the distribution of each of the studied variables was confirmed based on the Shapiro–Wilko test. One-way analysis of variance (ANOVA) was performed to determine the significance of differences between the variables. The homogeneity of variance in the groups was assessed using the Levene test. The Tukey RIR test was used to determine the significance of differences between the analyzed variables. The level of significance assumed in the tests was *p* = 0.05.

## 4. Conclusions

In this study, we investigated the use of post-fermentation leachate from a biogas plant that uses stillage and maize silage, as a culture medium for *Chlorella vulgaris*. It was found that the use of the leachate does not affect the biological activity of the microalgae. Based on the DPPH assay, the antioxidant activity of hydrophilic extracts from *Chlorella* sp. biomass were in the range of 2%–7%, which corresponds to 6%–21% of the activity of ascorbic acid. A significant increase in antioxidant activity was observed after ultrafiltration on filters with a cutoff of 50, 10 and 5 kDa. Antioxidant activity rose to up to about 20%, a level corresponding to 61% of the ascorbic acid activity. A similar relationship was observed for the protein fraction obtained as a result of precipitation with TCA followed by ultrafiltration. In this case, the highest antioxidant activity was about 10%, which was 30% of the ascorbic acid activity. Antioxidant activity may be associated with the presence of polyphenols in aqueous extracts of *Chlorella vulgaris* biomass. Based on a test using Folin–Ciocalteu reagent, it was found that the average polyphenol content, calculated as a pyrogallol, was 0.77 mM.

Both the hydrophilic extract from *Chlorella vulgaris* biomass and the protein component of the hydrophilic fraction were characterized by satisfactory antimicrobial activity. The inhibition zone assay showed that the aqueous extract of *Chlorella vulgaris* biomass is significantly more active against fungi (diameters of the inhibition zones was about 50 mm) compared to bacteria (diameters of the inhibition zones was about 25 mm). Based on an MIC test it has been found that the protein fraction has higher antimicrobial activity compared to a full aqueous extract, which may contain chemically diverse groups of hydrophilic compounds. The MICs were as follows: 16.25 mg/mL for *L. Plantarum* and *S. epidermidis*, 32.5 mg/mL for *C. albicans* and *S. cerevisiae*. The lowest activity was observed for *E. coli*, with MICs in the range of 32.5–65 mg/mL. The aqueous extract of *Chlorella vulgaris* biomass was also found to have activity against parasitic nematodes. At concentrations of 37.5, 75 and 150 mg/mL, the aqueous extract of *Chlorella vulgaris* biomass caused complete mortality of the nematode *Steinernema feltiae*.

The biological activity of the hydrophilic and protein fractions of *Chlorella vulgaris* biomass indicates that this microalgae could be successfully used as a source of natural antioxidant, antibacterial and antifungal compounds in food supplements and feed, as well as in natural methods for plant protection. Further research is underway on the isolation and characterization of the individual compounds responsible for this biological activity from the hydrophilic extract.

## Figures and Tables

**Table 1:** Summary of Lowry test results and total polyphenol contents.

Sample	Concentration of Tested Samples [mg/mL H_2_O]	Polyphenol Content (Folin–Ciocalteu Test) [mM] *
S1—water extraction, first repetition	27.0	0.77
S2—water extraction, second repetition	21.5	0.84
S3—water extraction, third repetition	20.6	0.71
S4—protein fraction obtained from S3 treated with TCA and then centrifuged once	18.8	0.33
S5—protein fraction obtained from S3 treated with TCA and then after isolation of S4. The supernatant was subjected to five centrifugations	19.6	0.16

* – match the pyrogaroll concentration

**Table 2:** Minimal inhibitory concentration (MIC) of the aqueous extract of *Chlorella* sp. biomass and the protein fraction against various microorganisms.

MIC [mg/mL]	*L. plantarum*	*S. epidermidis*	*E. coli*	*C. albicans*	*S. cerevisiae*
water extract	37.5–75	150	150	37.5–75	lack of activity
protein fraction precipitated by TCA, pellet obtained by a single centrifugation	16.25	16.25	32.5–65	32.5	32.50

**Table 3:** Characteristics of the post-fermentation leachate.

Parameters	Unit	Effluent	
		Crude	After Distillation
Dry Mass (DM)	mg/L	12740 ± 260	21 ± 4
Chemical Oxygen Demand	mg O_2_/L	8190 ± 170	1370 ± 20
Biochemical Oxygen Demand	mg O_2_/L	3960 ± 90	460 ± 30
N_total_	mg N/L	3710 ± 100	2150 ± 110
N-NH_4_	mg N-NH_4_/L	2540 ± 70	1450 ± 40
P_total_	mg P/L	350 ± 20	6.0 ± 2.0
P-PO_4_	mg P-PO_4_/L	240 ± 10	4.0 ± 1.0
pH	-	6.9 ± 0.4	7.1 ± 0.1

## References

[1] Priyadarshani I., Rath B. (2012). Commercial and industrial applications of micro algae —A review. J. Algal Biomass Utln..

[2] Wajahatullah K., Rayirath U.P., Subramanian S., Jithesh M.N., Prasanth R., Mark H.D., Alan C.T., James C.S., Jeff N., Balakrishan P. (2009). Seaweed Extracts as Biostimulants of Plant Growth and Development. J. Plant. Growth. Regul..

[3] Garson J. (1989). Marine Natural Products. Nat. Prod. Rep..

[4] Saleh B., Al-Mariri A. (2017). Antimicrobial Activity of the Marine Algal Extracts against Selected Pathogens. J. Agr. Sci. Tech..

[5] Sode S., Bruhn A., Balsby T.J.S., Larsen M.M., Gotfredsen A., Rasmussen M.B. (2013). Bioremediation of Reject Water from Anaerobically Digested Waste Water Sludge with Macroalgae (*Ulva lactuca*, Chlorophyta). Bioresour. Technol..

[6] Oumaskour K., Boujaber N., Etahiri S., Assobhel O. (2013). Anti-Inflammatory and Antimicrobial Activities of Twenty-Three Marine Algae from the Coast of SidiBouzid (El Jadida-Morocco). Int. J. Pharm. Pharm. Sci..

[7] Kausalya M., Rao G.M.N. (2015). Antimicrobial Activity of Marine Algae. J. Algal Biomass Utln..

[8] Zbakh H., Chiheb H., Bouziane H., Sánchez V.M., Riadi H. (2012). Antibacterial Activity of Benthic Marine Algae Extracts from the Mediterranean Coast of Morocco. J. Microb. Biotech. Food Sci..

[9] Malingin D.L., Hongayo M.C., Larino R.C. (2012). Antibacterial and Antioxidant Effects of Brown Alga *Padina australis* Hauck Crude Extract. IAMURE Int. J. Sci. Clin. Lab..

[10] Jeyaseelan E.C., Kothai S., Kavitha R., Tharmila S., Thavaranjit A.C. (2012). Antibacterial Activity of Some Selected Algae Present in the Costal Lines of Jaffna Peninsula. Int. J. Pharm. Biol. Arch..

[11] Alghazeer R., Whida F., Abduelrhman E., Gammoudi F., Azwai S. (2013). Screening of Antibacterial Activity in Marine Green, Red and Brown Macroalgae from the Western Coast of Libya. Nat. Sci..

[12] Abo-State M.A.M., Shanab S.M.M., Ali H.E.A., Abdullah M.A. (2015). Screening of Antimicrobial Activity of Selected Egyptian Cyanobacterial Species. J. Ecol. Health Environ..

[13] Karabay-Yavasoglu N.U., Sukatar A., Ozdemir G., Horzum Z. (2007). Antimicrobial Activity of Volatile Components and Various Extracts of the Red Alga Janiarubens. Phytother. Res..

[14] Abd El-Hack M.E., Abdelnourm S., Alagawany M., Abdo M., Sakr M.A., Khafaga A.F., Mahgoub S.A., Elnesr S.S., Gebriel M.G. (2019). Microalgae in modern cancer therapy: Current knowledge. Biomed. Pharmacother..

[15] Martínez Andrade K.A., Lauritano C., Romano G., Ianora A. (2018). Marine Microalgae with Anti-Cancer Properties. Mar. Drugs.

[16] Lauritano C., Andersen J.H., Hansen E., Albrigtsen M., Escalera L., Esposito F., Helland K., Hanssen K.Ø., Romano G., Ianora A. (2018). Bioactivity Screening of Microalgae for Antioxidant, Anti-Inflammatory, Anticancer, Anti-Diabetes, and Antibacterial Activities. Front. Mar. Sci..

[17] Kang K.H., Kim S.K. (2013). Beneficial effect of peptides from microalgae on anticancer. Curr. Protein Pept. Sci..

[18] Villarruel-López A., Ascencio F., Nuño K. (2017). Microalgae, a Potential Natural Functional Food Source —A Review. Pol. J. Food Nutr. Sci..

[19] Chu W.L. (2012). Biotechnological applications of microalgae. IeJSME.

[20] Parmar A., Singh N.K., Pandey A., Gnansounou E., Madamwar D. (2011). Cyanobacteria and microalgae: A positive prospect for biofuels. Bioresour. Technol..

[21] Mostafa S.S.M. (2012). Microalgal Biotechnology: Prospects and Applications. Plant. Sci..

[22] Ibañez E., Cifuentes A. (2013). Benefits of using algae as natural sources of functional ingredients. J. Sci. Food Agric..

[23] Batista A.P., Gouveia L., Bandarra N.M., Franco J.M., Raymundo A. (2013). Comparison of microalgal biomass profiles as novel functional ingredient for food products. Algal Res..

[24] Safi C., Zebib B., Merah O., Pontalier P.Y., Vaca-Garcia C. (2014). Morphology, composition, production, processing and applications of Chlorella vulgaris: A review. Renew. Sust. Energ. Rev..

[25] Fradique M., Batista A.P., Nunes M.C., Gouveia L., Bandarra N.M., Raymundo A. (2010). Incorporation of *Chlorella vulgaris* and *Spirulina maxima* biomass in pasta products. Part 1: Preparation and evaluation. J. Sci. Food Agric..

[26] Li H.-B., Jiang Y., Chen F. (2002). Isolation and purification of lutein from the microalga *Chlorella vulgaris* by extraction after saponification. J. Agric. Food Chem..

[27] Fernandes B., Dragone G., Abreu A., Geada P., Teixeira J., Vicente A. (2012). Starch determination in *Chlorella vulgaris* – a comparison between acid and enzymatic methods. J. Appl. Phycol..

[28] Grobbelaar J.U. (2003). Quality Control and Assurance: Crucial for the sustainability of the applied phycology industry. J. Appl. Phycol..

[29] Gulati O.P., Berry Ottaway P. (2006). Legislation relating to nutraceuticals in the European Union with a particular focus on botanical-sourced products. Toxicology.

[30] Rodriguez-Garcia I., Guil-Guerrero J.L. (2008). Evaluation of the antioxidant activity of three microalgal species for use as dietary supplements and in the preservation of foods. Food Chem..

[31] Gouveia L., Oliveira C. (2009). Microalgae as a raw material for biofuels production. J. Ind. Microbiol. Biotechnol..

[32] Miranda J.R., Passarinho P.C., Gouveia L. (2012). Bioethanol production from *Scenedesmus obliquus* sugars: The influence of photobioreactors and culture conditions on biomass production. Appl. Microbiol. Biotechnol..

[33] Marques A.E., Barbosa T.A., Jotta J., Tamagnini P., Gouveia L. (2011). Biohydrogen production by *Anabaena* sp. PCC 7120 wild-type and mutants under different conditions: Light, Nickel and CO_2_. Biomass Bioenerg..

[34] Ferreira A.F., Ribeiro L., Batista A.P., Marques P.A.S.S., Nobre B.P., Silva P.P., Gouveia L., Silva C. (2013). A Biorefinery from *Nannochloropsis* sp. microalga —Energy and CO_2_ emission and economic analyses. Bioresour. Technol..

[35] Batista A.P., Moura P., Marques P.A.S.S., Ortigueira J., Alves L.M., Gouveia L. (2014). *Scenedesmus obliquus* as a feedstock for bio-hydrogen production by *Enterobacter aerogenes* and *Clostridium butyricum* by dark fermentation. Fuel.

[36] Gouveia L. (2014). From Tiny Microalgae to Huge Biorefineries. Oceanography.

[37] Allen J., Unlu S., Demirel Y., Black P., Riekhof W. (2018). Integration of biology, ecology and engineering for sustainable algal-based biofuel and bioproduct biorefinery. Bioresour. Bioprocess..

[38] Schiano di Visconte G., Spicer A., Chuck C.J., Allen M.J. (2019). The Microalgae Biorefinery: A Perspective on the Current Status and Future Opportunities Using Genetic Modification. Appl. Sci..

[39] Koyande A.K., Show P.-L., Guo R., Tang B., Ogino C., Chang J.-S. (2019). Bio-processing of algal bio-refinery: A review on current advances and future perspectives. Bioengineered..

[40] Laurens L.M.L., Markham J., Templeton D.W., Christensen E.D., Van Wychen S., Vadelius E.W., Chen-Glasser M., Dong T., Davis R., Pienkos P.T. (2017). Development of algae biorefinery concepts for biofuels and bioproducts; a perspective on process-compatible products and their impact on cost-reduction. Energy Environ. Sci..

[41] Kolesinska B., Fraczyk J., Binczarski M., Modelska M., Berlowska J., Dziugan P., Antolak H., Kaminski Z.J., Witonska I.A., Kregiel D. (2019). Butanol Synthesis Routes for Biofuel Production: Trends and Perspectives. Materials.

[42] Collet P., Hélias A., Lardon L., Ras M., Goy R.A., Steyer J.P. (2011). Lifecycle assessment of microalgae culture coupled to biogas production. Bioresour. Technol..

[43] Ehimen E.A., Sun Z.F., Carrington C.G., Birch E.J., Eaton-Rye J.J. (2011). Anaerobic digestion of microalgae residues resulting from the biodiesel production process. Appl. Energ..

[44] Gouveia L., Neves C., Sebastião D., Nobre B.P., Matos C.T. (2014). Effect of light on the production of bioelectricity and pigments by a Photosynthetic Alga Microbial Fuel Cell. Bioresour. Technol..

[45] Powel E.E., Hill G.A. (2009). Economic assessment of an integrated bioethanol-biodiesel-microbial fuel cell facility utilizing yeast and photosynthetic algae. Chem. Eng. Res. Design.

[46] Campenni’ L., Nobre B.P., Santos C.A., Oliveira A.C., Aires-Barros M.R., Palavra A.M., Gouveia L. (2013). Carotenoids and lipids production of autotrophic microalga *Chlorella protothecoides* under nutritional, salinity and luminosity stress conditions. Appl. Microbiol. Biotechnol..

[47] Mussgnug J.H., Klassen V., Schlüter A., Kruse O. (2010). Microalgae as substrates for fermentative biogas production in a combined biorefinery concept. J. Biotechnol..

[48] Tsao R. (2010). Chemistry and Biochemistry of Dietary Polyphenols. Nutrients.

[49] Acharya M.M., Katyare S.S. (2004). An Improved Micromethod for Tyrosine Estimation. Z. Naturforsch..

[50] Barbarino E., Louren S.O. (2005). An evaluation of methods for extraction and quantification of protein from marine macro- and microalgae. J. Appl. Phycol..

[51] Becker E.W. (2007). Micro-algae as a source of protein. Biotechnol. Adv..

[52] Safi C., Charton M., Pignolet O., Silvestre F., Vaca-Garcia C., Pontalier P.-Y. (2013). Influence of microalgae cell wall characteristics on protein extractability and determination of nitrogen-to-protein conversion factors. J. Appl. Phycol..

[53] Berliner M.D. (1986). Proteins in *Chlorella vulgaris*. Microbios.

[54] Vehapi M., Koçer A.T., Yılmaz A., Özçimen D. (2019). Investigation of the antifungal effects of algal extracts on apple-infecting fungi. Arch. Microbiol..

[55] Selivanova E.A., Ignatenko M.E., Nemtseva N.V. (2014). Antagonistic activity of novel green microalgae strain. Zh. Mikrobiol. Epidemiol. Immunobiol..

[56] Pina-Pérez M.C., Rivas A., Martínez A., Rodrigo D. (2017). Antimicrobial potential of macro and microalgae against pathogenic and spoilage microorganisms in food. Food Chem..

[57] Mudimu O., Rybalka N., Bauersachs T., Born J., Friedl T., Schulz R. (2014). Biotechnological Screening of Microalgal and Cyanobacterial Strains for Biogas Production and Antibacterial and Antifungal Effects. Metabolites.

[58] Ghasemi Y., Moradian A., Mohagheghzadeh A., Shokravi S., Morowvat M.H. (2007). Antifungal and antibacterial activity of the microalgae collected from paddy fields of Iran: Characterization of Antimicrobial Activity of Chroococcus dipersus. J. Biol. Sci..

[59] Gaugler R., Lewis E., Stuart R.J. (1997). Ecology in the service of biological control: The case of entomopathogenic nematodes. Oecologia.

[60] Choleva B., Bileva T., Tzvetkov Y., Barakov P. (2005). Preliminary study of the green algae chlorella (*Chlorella vulgaris*) for control on the root-knot nematode (*Meloidogyne arenaria*) in tomato plants and ectoparasite *Xiphinema indexin* grape seedlings. Commun. Agric. Appl. Biol. Sci..

[61] Debowski M., Szwaja S., Zielinski M., Kisielewska M., Stanczyk-Mazanek E. (2017). The Influence of Anaerobic Digestion Effluents (ADEs) Used as the Nutrient Sources for Chlorella sp. Cultivation on Fermentative Biogas Production. Waste Biomass Valori..

[62] Debowski M., Rusanowska P., Zielinski M., Dudek M., Romanowska-Duda Z. (2018). Biomass production and nutrient removal by Chlorella vulgaris from anaerobic digestion effluents. Energies.

[63] Zielinski M., Debowski M., Szwaja S., Kisielewska M. (2018). Anaerobic Digestion Effluents (ADEs) Treatment Coupling with Chlorella sp. Microalgae Production. Water Environ. Res..

[64] Lowry O.H., Rosebrough N.J., Farr A.L., Randall R.J. (1951). Protein measurement with the Folin-phenol reagent. J. Biol. Chem..

[65] Agbor G.A., Vinson J.A., Donnelly P.E. (2014). Folin-Ciocalteau Reagent for Polyphenolic Assay. Int. J. Food Sci. Nutr. Diet..

[66] Zych I., Krzepiłko A. (2010). Measurement of total antioxidant capacity of selected antioxidants and infusions using DPPH radical reduction. Chem.-Didact.-Ecol.-Metrol..

